# Sex-Specific Neuropsychiatric Effects of Subanesthetic Ketamine Exposure in Pregnant Mice and Their Offspring

**DOI:** 10.1007/s10571-025-01582-w

**Published:** 2025-07-19

**Authors:** Wei-Sheng Lin, Pei-Yu Wang, Sheng-Rong Yeh, Zoe Lai, Andrew Chengyu Lee, Shou-Zen Fan

**Affiliations:** 1 Department of Pediatrics, Taipei Veterans General Hospital, Taipei, Taiwan; 2 School of Medicine, National Yang Ming Chiao Tung University, Taipei, Taiwan; 3 Graduate Institute of Brain and Mind Sciences, College of Medicine, National Taiwan University, Taipei, Taiwan; 4 Neurobiology and Cognitive Science Center, National Taiwan University, Taipei, Taiwan; 5 Center for Systems Biology, National Taiwan University, Taipei, Taiwan; 6 Ph.D. Program in Translational Medicine, National Taiwan University and Academia Sinica, Taipei, Taiwan; 7 Graduate Institute of Neural Regenerative Medicine, College of Medical Science and Technology, Taipei Medical University, Taipei, Taiwan; 8 Department of Anesthesiology, En Chu Kong Hospital, 399, Fuxing Rd., Sanxia Dist., New Taipei City, 237414 Taiwan; 9 Department of Anesthesiology, School of Medicine, National Taiwan University, Taipei, Taiwan

**Keywords:** Anxiety, Depression, Hippocampus, N-methyl-D-aspartate receptor, Ketamine

## Abstract

Depression during pregnancy is often overlooked and undertreated. Ketamine has been shown to exert prompt and sustained antidepressant effects in patients with depression, although concerns of potential neurotoxicity prohibit its use in pregnant women. Here, we aim to investigate the neurobehavioral effects of subanesthetic ketamine on pregnant mice and their offspring. We found that pregnant C57BL/6 mice receiving ketamine (10 mg/kg/day intraperitoneal) from gestation day 15 to 17 exhibited less depression-like behaviors. Prenatal ketamine treatment induced male-specific reduction in depression- and anxiety-like behaviors in adult offspring, without alterations in social and memory performance. These behavioral outcomes were associated with a male-specific increase in dendritic spine density of dentate gyrus granule cells, while neither dendritic architecture nor hippocampal neurogenesis was affected. N-methyl-D-aspartate receptor subunits GluN2A and GluN3A were expressed at significantly higher levels in the hippocampus of male as compared to female mouse embryos, suggesting sex-dependent actions of ketamine on developing brain. Overall, our study showed that prenatal exposure to subanesthetic ketamine could exert long-lasting neurobehavioral effects in a sex-dependent manner, with male offspring being more resilient to stress. These findings may have implications concerning ketamine use during pregnancy, and also provide clues about the developmental origins of emotional problems.

**Supplementary Information:**

The online version contains supplementary material available at 10.1007/s10571-025-01582-w.

## Background

Major depressive disorder is a common and disabling condition, with a lifetime prevalence of up to ~ 20% (Hasin et al. 2018). Depression has been ranked as the third leading contributor to the global disease burden, with significant socioeconomic impacts (Collins et al. 2011; Greenberg et al. 2015). It is also an important issue in childbearing women (Li et al. 2024; Stewart 2011; Vigod et al. 2016). Indeed, depression during pregnancy poses unique therapeutic challenges (Chaudron 2013), and has been associated with adverse pregnancy and neonatal outcomes (Grigoriadis et al. 2013).

Although antidepressant medications are often prescribed, their use remains controversial during pregnancy (Campagne 2018). Issues of concern include potential teratogenicity and long-term effects of antidepressants on the offspring, which have been actively investigated in recent years (Stewart 2011; Huybrechts et al. 2014; Brown et al. 2016; Vigod et al. 2016; Campagne 2018; Uguz 2018; Singal et al. 2020). Moreover, conventional antidepressants often have a delayed onset, with effects emerging weeks after treatment begins, and many patients remain unresponsive to these medications, which mainly target the serotonergic system. Substantial evidence indicates that ketamine at a subanesthetic dose has a rapid-onset (i.e., within hours) antidepressant effect in humans (Berman et al. 2000; Zarate et al. 2006; Murrough et al. 2013) and rodents (Autry et al. 2011; Zanos et al. 2016; Yang et al. 2018). However, prescribing ketamine to pregnant women with depression is not recommended because limited information is available about its effects on the offspring. Ketamine is a noncompetitive N-methyl-D-aspartate receptor (NMDAR) antagonist that readily crosses the placenta (Little et al. 1972; Ellingson et al. 1977). NMDAR antagonists may trigger neuronal apoptosis and/or pyroptosis in developing brain in rodents and nonhuman primates (Ikonomidou et al. 1999; Brambrink et al. 2012; Zhang et al. 2021). In a series of rat studies, maternal exposure to sedative-dose ketamine during middle gestation (E14) also resulted in widespread apoptosis in fetal brain, as well as neuronal loss and altered dendritic branching in prefrontal cortex in the offspring (Zhao et al. 2016). Memory impairment and depression-like behavior were observed in these offspring at young age (Zhao et al. 2014). In vitro experiments with rat neural stem progenitor cells suggested that ketamine could affect neuronal proliferation and differentiation (Dong et al. 2012). Research in rhesus macaques suggests that perinatal ketamine exposure is potentially neurotoxic and is associated with long-lasting neurocognitive disturbance and decreased expressions of emotionality (Paule et al. 2011; Brambrink et al. 2012, Capitanio et al. 2012). On the other hand, the adverse neurodevelopmental effects of ketamine seem to be developmental stage-dependent, and they appear most prominent during late fetal to early neonatal period in rats (Ikonomidou et al. 1999; Mickley et al. 2001; Walker et al. 2010). The level and time course of drug exposure may also be relevant, and it remains to be clarified whether maternal ketamine exposure at a lower (i.e., antidepressant-equivalent) dose is detrimental to the neurodevelopment of offspring. Motivated by the aforementioned knowledge gaps, we examined the neuronal and behavioral effects of subanesthetic ketamine on pregnant mice and their offspring in the present study. Unexpectedly, we found that subanesthetic ketamine treatment during pregnancy was associated with enhanced stress resilience in male offspring.

## Materials and Methods

### Animals

**Fig. 1:**
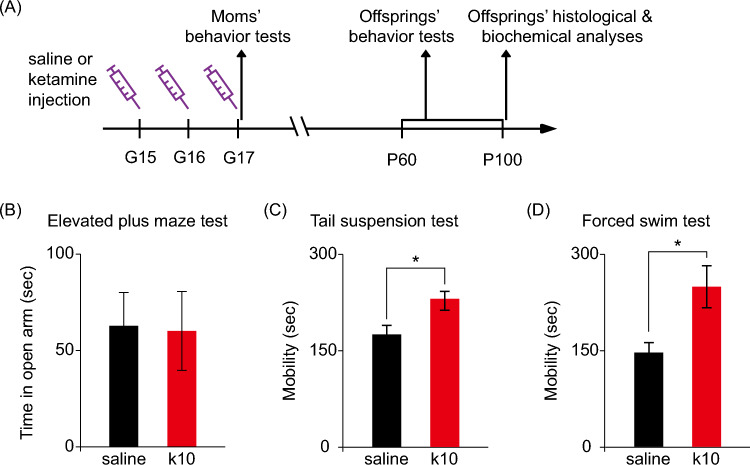
Subanesthetic ketamine treatment induces antidepressant-like effects in pregnant mice. **A** The experimental workflow: pregnant mice received daily intraperitoneal injections of either saline or 10 mg/kg ketamine (k10) from gestation (G) day 15 to 17. The offspring were collected for the subsequent behavioral, histological and biochemical analyses from postnatal day (P) 60 to 100. **B**–**D** Pregnant mice were subjected to elevated plus maze test (**B**), tail suspension test (**C**), and forced swim test (**D**) at G17. Data are presented as mean ± SEM (*n* = 6 mice per group). *P < 0.05 by Student’s t test

All experimental procedures followed local animal ethics regulations and were approved by National Taiwan University College of Medicine and College of Public Health Institutional Animal Care and Use Committee (Approval No: 20190079). Pregnant C57BL/6 mice were obtained from BioLASCO Taiwan Co., Ltd. Mice were maintained in an animal room with controlled temperature of 22–24 °C and humidity of 50–55%, under a 12-h light/dark cycle. Either subanesthetic ketamine (10 mg/kg, Sigma) or saline was injected intraperitoneally into pregnant mice once per day from gestation day 15 to 17 at roughly the same time each day (Fig. 1A). Ketamine 10 mg/kg is the most frequently used dose in rodent depression-related research based on an extensive review of previous studies (Polis et al. 2019). The pregnant mice were then subjected to behavioral analysis on gestation day 17, in the order of elevated plus maze test, tail suspension test, and forced swim test, with an interval of ~ 1 h between different behavioral tests. Food intake and body weight of the offspring were monitored regularly. All of the subsequent behavioral, histological, and biochemical analyses were done blinded with respect to the sex and treatment of the offspring mice. A panel of behavioral analyses was performed during postnatal day 60 to 100 (Fig. 1A). The sequence of behavioral tests was as follows: novel object recognition test, elevated plus maze test, three-chamber social test, tail suspension test, and forced swim test, with an interval of 1 day between different behavioral tests. All behavioral tests were conducted in the dark phase. The offspring mice were then sacrificed at postnatal day 100, and their brains were harvested for further histological and biochemical analyses (detailed in “Golgi Staining, Nissl Staining, and Dendritic Analyses” and Immunohistochemistry sections). All behavioral testing and subsequent analyses were conducted with the experimenters blinded to the treatment groups.

### Elevated Plus Maze Test

We used the elevated plus maze to evaluate the anxiety-like behavior of mice (Walf and Frye 2007). The maze consisted of a square central area (6 cm × 6 cm) and four arms (6 cm × 40 cm), with two open arms and two closed arms having 25.5 cm high walls. The animals were acclimated to the testing environment for ~ 30 min before starting the test. During the test, the mouse was placed on the center area and was allowed to explore the maze freely for 5 min. The time each mouse spent in open arms was recorded.

### Tail Suspension and Forced Swim Tests

The depression-like behaviors of mice were examined using the tail suspension and forced swim tests as described previously (Fan et al. 2021). Briefly, the animals were acclimated to the testing environment for ~ 30 min before starting the test. For the tail suspension test, each mouse was suspended from its tail and videotaped for 6 min. The mobility time of each individual mouse was recorded. To conduct the forced swim test, each mouse was placed in a water-containing cylinder (with a height of 30 cm and a diameter of 20 cm) for 6 min. The mouse would struggle in the water for some time, and the mobility time during the procedure was recorded.

### Three-Chamber Social Test

The three-chamber social test was performed as previously described (Fan et al. 2021). Briefly, the test mouse was first habituated for 30 min in a three-chambered box with transparent walls and access doors connecting the three chambers. For the subsequent sociability test, the mouse was put in the central chamber and allowed to freely explore the apparatus for 5 min, with a new mouse (A) enclosed in a cage placed in the left chamber, and an empty cage (E) in the right chamber. Immediately afterward, another novel mouse (B) was put into the empty cage and the test mouse was allowed to explore the apparatus freely again for 5 min. The time each test mouse spent interacting with E, A, and B was recorded, and the discrimination index (DI) for sociability and social novelty was calculated using the formulae (A − E)/(A + E) × 100 and (B − A)/(B + A) × 100, respectively.

### Novel Object Recognition Test

The novel object recognition test was performed as described previously (Teng et al. 2019). Briefly, individual mouse was exposed to two identical objects in an open arena (40 cm × 40 cm × 40 cm) for 30 min. Twenty-four hours later, the mouse was reintroduced into the same open arena for 30 min, with one of the familiar objects having been replaced by a novel object. We calculated the memory index of mice by subtracting the time spent on exploring the familiar object from the time spent on exploring the novel object, which was then divided by total time spent exploring both objects.

### Golgi Staining, Nissl Staining, and Dendritic Analyses

Morphological analyses of granule cells in the dentate gyrus (DG) were visualized using the FD Rapid GolgiStain™ kit following the manufacturer’s protocol (FD NeuroTechnologies). Dendritic morphology was reconstructed and analyzed using Neurolucida software (MBF, Bioscience), as described in our previous study (Teng et al. 2019). To avoid any sampling bias, we employed a systematic random sampling strategy for selecting sections for analysis. Specifically, for each mouse brain we collected serial coronal sections through the hippocampus and randomly selected equivalent sections from the ketamine group and control group for Golgi staining and analysis. Within each selected section, we included all well-impregnated granule cells that met our inclusion criteria (fully intact dendritic arbor in the plane of section). Typically, 3–5 granule cells per mouse (from non-adjacent sections spanning the dorsal hippocampus) were reconstructed. To analyze the cell density of DG granule cells, hippocampal sections of mouse brain were stained with 0.1% cresyl violet, and then examined using a 63 × oil-immersion objective lens on a photomicroscope (Zeiss Axio Imager 2). The number of DG granule cells was manually counted, and the contour of DG was depicted to determine its area using ImageJ software. The density of DG granule cells was then obtained through dividing the cell number by the area of DG.

### Immunohistochemistry

Mice were sacrificed by cardiac perfusion with 4% paraformaldehyde (Sigma), and brain tissues were embedded in FSC 22 Clear Frozen Section Compound (Leica). Brain sections were cut on a cryostat (Leica CM3050), and immunohistochemistry was performed as described previously (Wang et al. 2005; Chou et al. 2013). Antibodies included goat anti-doublecortin (1:250, Santa Cruz sc-8066), rabbit anti-Ki67 (1:500, Abcam ab15580), and donkey anti-goat and donkey anti-rabbit biotinylated secondary antibody (1:200, Jackson ImmunoResearch). Cy3-conjugated streptavidin (1:200, Jackson ImmunoResearch) and 4′,6-diamidino-2-phenylindole dihydrochloride (DAPI, SouthernBiotech) were used for labeling the immunoreactivity and cellular nuclei.

### mRNA Quantification

Total RNA was isolated from embryonic (gestation day 17) hippocampal tissue of mice unexposed to ketamine using the NucleoSpin RNA Kit (Macherey–Nagel), and cDNA was prepared using oligo-d(T)_15_ (Invitrogen) and SuperScript III reverse transcriptase (Invitrogen), as described previously (Huang et al. 2015). Quantitative PCR was performed using a StepOnePlus Real-Time PCR System (Applied Biosystems), with SYBR Green Master Mix (Fermentas) and gene-specific primers (Table S1). *Tubb* (encoding β-Tubulin) was used as the control gene. We confirmed the stability of *Tubb* expression by observing consistent Ct values across all samples. As previously described, a two-step PCR reaction was carried out with cycles of denaturation at 95 °C for 15 s and then annealing and extension combined at 60 °C for 1 min. The mRNA expression level of each target gene compared with *Tubb* was quantified by subtraction: Ct (specific gene) − Ct (*Tubb*) = ΔCt. A difference of one PCR cycle equates to a twofold change in mRNA expression level. The uniqueness of amplicons was confirmed using dissociation curves.

### Statistical Analysis

All data are expressed as mean ± SEM and were compared using Student’s t test or two-way analysis of variance (ANOVA) when appropriate. Fisher's Least Significant Difference (LSD) post hoc test was performed for pairwise comparisons if needed. All statistical tests were two-tailed, and the significance level was set at *P* < 0.05. Statistically significant findings were marked with an asterisk (*) in the Figures.

## Results

### Subanesthetic Ketamine Treatment During Late Gestation Induced Antidepressant-Like Effects in Pregnant Mice

Time-mated female mice were divided into two groups (n = 6 for each group), the vehicle control group and the subanesthetic ketamine (10 mg/kg) group. Both groups of mice received daily intraperitoneal injections of vehicle or ketamine from gestational day 15 to day 17 (Fig. 1A). Behavioral testing of pregnant mice showed no difference in elevated plus maze test between ketamine-treated and control groups, suggesting normal anxiety-like behavior in these pregnant mice (Fig. 1B). In contrast, ketamine-treated pregnant mice had longer mobility time in the antidepressant-predictive tasks, namely tail suspension test and forced swim test (Fig. 1C and D). These data agreed with previous reports showing that ketamine acts as an effective antidepressant in rodent models (Autry et al. 2011; Zanos et al. 2016; Yang et al. 2018).

### Subanesthetic Ketamine Treatment in Late Gestation Induced Reduced Body Weight in the Offspring

**Fig. 2:**
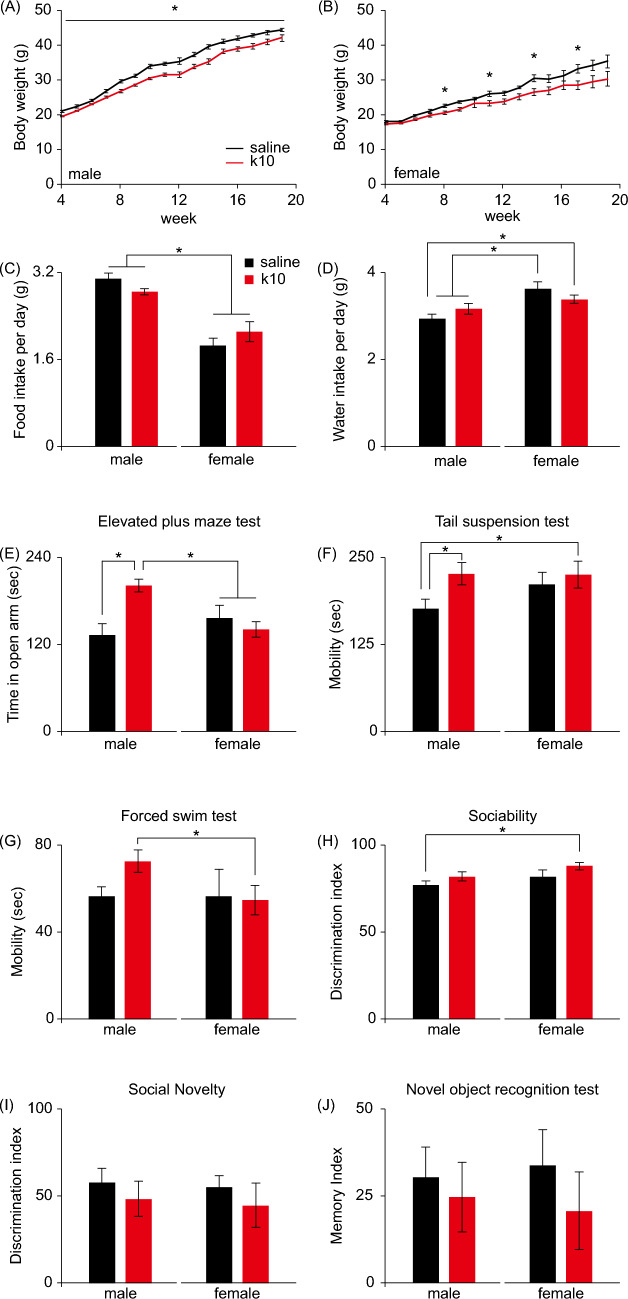
Subanesthetic ketamine treatment during late gestation induces male offspring-specific behavioral alterations. **A**, **B** The body weight of male (**A**) and female (**B**) offspring of pregnant mice receiving either saline or 10 mg/kg ketamine (k10) treatment daily during gestation day 15–17. **C** The food intake of male and female offspring of k10-treated pregnant mice. **D** The water intake of male and female offspring of k10-treated pregnant mice. **E**–**J** The neuropsychiatric status of the offspring of k10-treated pregnant mice were evaluated using a battery of behavioral tests, including elevated plus maze test (**E**), tail suspension test (**F**), forced swim test (**G**), three-chamber social test (**H** and **I**), and novel object recognition test (**J**). Data are presented as mean ± SEM (*n* = 6–15 mice per group). *P < 0.05, two-way ANOVA followed by Fisher’s LSD post hoc test

The number of offspring mice was 18 (6 females, 12 males) in control group and 26 (11 females, 15 males) in maternal ketamine treatment group. At 4 weeks of age, we found that male offspring of pregnant mice that had received ketamine showed significantly reduced body weight, whereas female offspring merely displayed a trend towards lower body weight, compared to control groups (Fig. 2A and B). The overall growth rate was lower for both adult male and female offspring in the ketamine groups (Fig. 2A and B), and the reduced body weight is not due to reduced adult food intake or water consumption (Fig. 2C and D).

### Subanesthetic Ketamine Treatment in Late Gestation Induced Male Offspring-Specific Behavioral Alterations

To evaluate the neurobehavioral effects of prenatal exposure to subanesthetic ketamine on the offspring, we conducted a panel of behavioral analyses during postnatal day 60 to 100 (Fig. 1A). Intriguingly, sex differences were found in behavioral tests relevant to anxiety and depression. Specifically, male offspring of ketamine-treated pregnant mice exhibited less anxiety- and depression-like behaviors in the elevated plus maze test (Fig. 2E) and tail suspension test (Fig. 2F), and also a marginal trend (*P* = 0.0794) toward less depression-like behavior in forced swim test (Fig. 2G). On the other hand, no differences were seen in female offspring of ketamine-treated versus control groups (Fig. 2E–G). Prenatal subanesthetic ketamine treatment did not affect sociability (novel mouse vs. empty cage) and social novelty (novel mouse vs. familiar mouse) of either male or female offspring in the three-chamber social test (Fig. 2H and I). We also found that the memory performance of both male and female offspring of ketamine-treated pregnant mice was comparable to that of the control groups in the 24-h novel object recognition test (Fig. 2J).

### Subanesthetic Ketamine Treatment During Late Gestation Induced Male Offspring-Specific Structural Alterations in Hippocampal Neurons

**Fig. 3:**
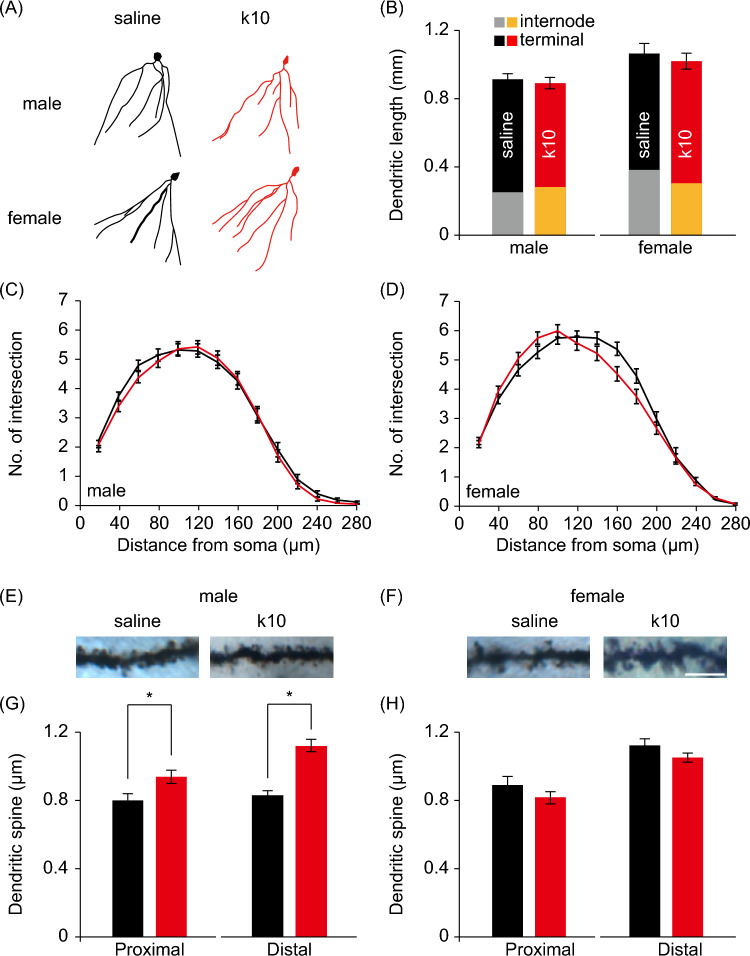
Subanesthetic ketamine treatment during late gestation induces male offspring-specific structural alterations in hippocampal neurons. Structural analyses of DG granule cells from male and female offspring of pregnant mice receiving either saline or 10 mg/kg ketamine (k10) treatment daily during gestation day 15–17. **A** Reconstructed DG granule cells. **B**–**D** The dendritic length (**B**) and dendritic profile (**C**, **D**) of granule cells were evaluated using the concentric-ring method of Sholl. **E**–**H** Representative images of distal dendrites (**E**, **F**; scale bar = 5 µm) and quantitative spine density (**G**, **H**) in distal (> 150 μm from the soma) and proximal (< 50 μm from the soma) dendrites of DG granule cells. Data are presented as mean ± SEM (*n* = 28–44 cells and 33–73 dendritic segments for each group). *P < 0.05 by Student’s t test or two-way ANOVA followed by Fisher's LSD post hoc test

Following the behavioral analyses, brains from each group were harvested, and morphological analyses of granule cells were performed using the Golgi-Cox impregnation method. We focused on hippocampal DG given its critical role in memory and emotional regulation (Yun et al. 2016). The morphologic features of DG granule cells in the offspring were reconstructed using Neurolucida software and subjected to Sholl analysis. No differences in dendritic architecture, as measured by dendritic length and complexity, were detected in the offspring between ketamine-treated groups and control groups (Fig. 3A–D; Fig. S1). However, male offspring of ketamine-treated pregnant mice exhibited higher dendritic spine density in both proximal and distal portions of dendrites, as compared to male offspring of control mice (Fig. 3E and G). No difference in dendritic spine density was found between female offspring of ketamine-treated versus control groups (Fig. 3F and H).

### Subanesthetic Ketamine Treatment During Late Gestation Did Not Affect Hippocampal Neurogenesis in the Offspring

**Fig. 4:**
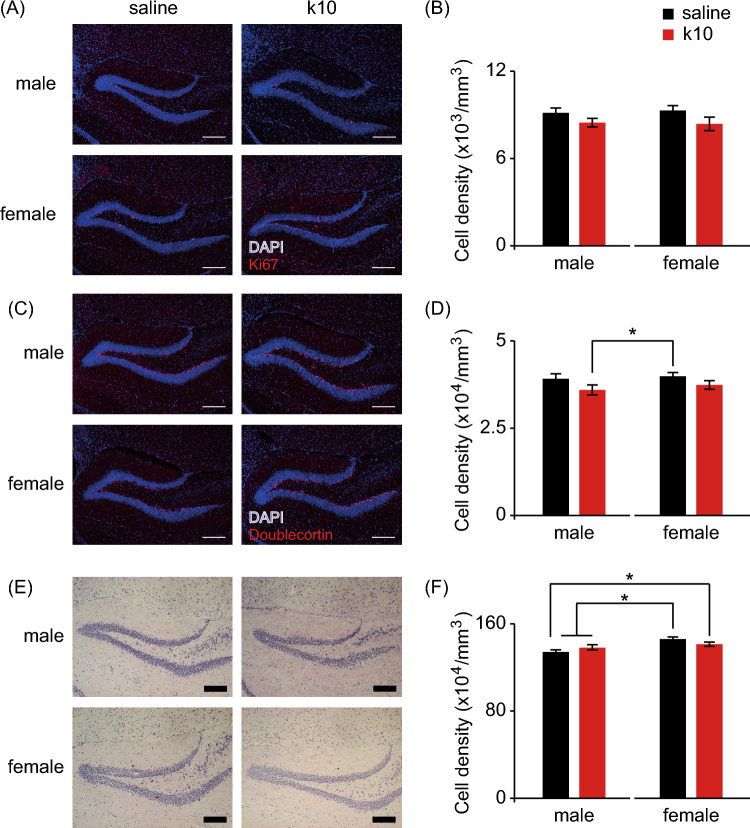
Subanesthetic ketamine treatment during late gestation does not affect hippocampal neurogenesis in mouse offspring. Histological analyses of DG from male and female offspring of pregnant mice receiving either saline or 10 mg/kg ketamine (k10) treatment daily during gestation day 15–17. Representative micrographs (**A**, **C**, **E**) and quantitative cell density (**B**, **D**, **F**) of proliferating cells (**A**, **B**), immature neurons (**C**, **D**) and granule cells (**E**, **F**) following immunostaining and Nissl staining. Anti-Ki67 antibody, anti-doublecortin antibody and DAPI were used to label proliferating cells, immature neurons and cellular nuclei, respectively. Data are presented as mean ± SEM (*n* = 64–114 DG regions for each group). Scale bar = 200 µm. *P < 0.05, two-way ANOVA followed by Fisher’s LSD post hoc test

Since increased adult hippocampal neurogenesis is required for the behavioral effect of anti-depressants in animals (Santarelli et al. 2003; David et al. 2009; Hill et al. 2015), we examined the neurogenesis activity in the DG of offspring of ketamine-treated pregnant mice. We used Ki67 and doublecortin as markers for cellular proliferation and immature neurons, respectively, and found that perinatal ketamine treatment did not affect hippocampal neurogenesis in either male or female offspring (Fig. 4A–D). Moreover, perinatal ketamine treatment did not change the granule cell density of the DG upon examination with Nissl staining (Fig. 4E and F).

### Sex Differences in NMDAR Subunit Expression During Neurodevelopment

**Fig. 5:**
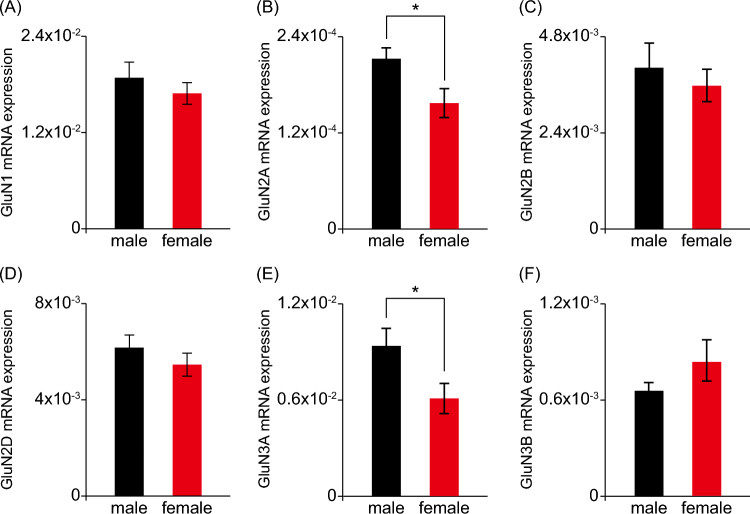
Sex differences in NMDA receptor subunit expression during development. Hippocampal tissues were obtained from gestation day 17 embryos, and mRNA expression levels of NMDA receptor subunits, including GluN1 (**A**), GluN2A (**B**), GluN2B (**C**), GluN2D (**D**), GluN3A (**E**), and GluN3B (**F**) were measured using qPCR. The sex of each embryo was determined by the expression of sex-determining region of Y chromosome (SRY) gene. Data are presented as mean ± SEM (*n* = 10 tissues for each group). *P < 0.05 by Student’s *t* test

Ketamine has been shown to act as an NMDAR antagonist, and NMDARs are assembled as heteromers with various combinations of subunits including GluN1, GluN2A, GluN2B, GluN2C, GluN2D, GluN3A and GluN3B (Paoletti et al. 2013). Since the subunit composition of NMDAR could dictate its properties, we hypothesized that differential expression of NMDAR subunits in embryonic brain between male and female may contribute to the observed male offspring-specific neuronal structural and behavioral alterations. We quantified the mRNA expression levels of each NMDAR subunit in hippocampal tissues of gestation day 17 embryos, and found significantly higher mRNA expression levels of GluN2A and GluN3A, but not GluN1, GluN2B, GluN2D, or GluN3B subunits, in the male embryonic hippocampal tissues compared to females (Fig. 5). GluN2C mRNA was not detected in our preparations, consistent with its postnatal expression pattern as observed in previous studies (Paoletti et al. 2013). The sex differences in glutamate receptor subunit expression may contribute to the sex-specific effects of prenatal ketamine exposure at behavioral (“Subanesthetic Ketamine Treatment in Late Gestation Induced Male Offspring-Specific Behavioral Alterations” section) and neuronal morphological (“Subanesthetic Ketamine Treatment During Late Gestation Induced Male Offspring-Specific Structural Alterations in Hippocampal Neurons” section) levels.

## Discussion

Our study reveals that subanesthetic-dose ketamine confers antidepressant-like effects in pregnant mice without compromising pregnancy outcomes, and that maternal exposure does not adversely affect offspring behavior in motor, cognitive, and social domains. The enhanced stress resilience observed in male offspring in our study contrasts sharply with previous reports highlighting ketamine’s developmental neurotoxicity and its long-term neurobehavioral consequences (Ikonomidou et al. 1999; Paule et al. 2011; Zhao et al. 2014, 2016; Kalopita et al. 2021). The discrepancy may be explained by various dosing schedules and time windows of ketamine administration in different experimental contexts (Wang 2012; Yan and Jiang 2014; Zhang et al. 2022). For instance, Aligny et al. administered a much larger dose of ketamine (50 mg/kg subcutaneous daily) to pregnant mice from gestation day 15 to parturition, and reported long-term impairments in cortical GABAergic interneuron integration and behavior in offspring (Aligny et al. 2014).

We found sex-specific effects of prenatal low-dose ketamine exposure at neuronal and behavioral levels, with male offspring exhibiting higher dendritic spine density in hippocampal neurons, and less anxiety- and depression-like behaviors in young adulthood. Although this male-specific antidepressant effect was considered to be significant only in tail suspension test (Fig. 2F), a similar trend could also be observed in forced swim test (Fig. 2G). It has been noted that forced swim test is less sensitive than tail suspension test in detecting drug response in some experimental contexts (Cryan et al. 2005), which could possibly explain the non-significant result in our study. Furthermore, we noted the sex difference in the expression of some NMDAR subunits in fetal mice, with higher expression of GluN2A and GluN3A in the hippocampus of male as compared to female. It is worth noting that differences in glutamate receptor gene expression between sexes have also been observed in patients with major depressive disorder (Gray et al. 2015). Taken together, these findings suggest that ketamine might act on the sex-specific NMDAR repertoire of embryonic brain, thereby resulting in long-lasting sexually dimorphic changes in mood-related glutamatergic pathways. This is compatible with the notion that mental disorders or stress vulnerability/resilience may stem from early life experiences (Su et al. 2021). On the other hand, gonadal hormones may also affect the propensity for depression, as reflected by the sucrose preference in animal models (Saland et al. 2016). Some gonadal hormone modulates glutamatergic neurotransmission in the hippocampus through sex-specific molecular mechanisms (Oberlander and Woolley 2016). It is interesting to note that antidepressant-like effect of ketamine was dependent on the presence of ovarian hormones in female mice according to a recent study (Chen et al. 2020). It remains to be elucidated whether the sex-dependent effects of prenatal ketamine exposure on emotional regulation in adult animals are related to the physiological elevation of gonadal hormones after pubertal onset.

Maternal exposure to sedative-dose ketamine has been associated with alterations in neuronal morphology in terms of dendritic branching pattern in rats at postnatal day 30 (Zhao et al. 2016). In contrast, we did not find significant differences in dendritic architecture in the offspring of subanesthetic-dose ketamine-treated pregnant mice at postnatal day 100 (Fig. 3A–D), while we found higher spine density in both proximal and distal dendrites in male offspring of ketamine-treated pregnant mice (Fig. 3E and G). Alterations in dendritic spine density have been observed in chronic stress models of depression, and dendritic spine formation appeared to correlate with antidepressant effect of ketamine (Li et al. 2010; Duman and Duman 2015; Krystal et al. 2023). Therefore, it is plausible to speculate that prenatal exposure to ketamine leads to increased dendritic spine formation in male mice, and the consequent higher dendritic spine density endows these male mice with stress-resilient behaviors in our study. Nonetheless, whether these are causally linked remains to be experimentally verified. Alternatively, the increased dendritic spine density in male offspring may reflect the adaptive capacity of these mice after being subjected to stressful conditions such as tail suspension or forced swimming in our experimental context. This explanation is in line with earlier reports suggesting a role for increased excitatory synapse formation in ameliorating behavioral despair, although previous studies have shown this in the prefrontal cortex (Li et al. 2010, 2011).

Despite the encouraging findings of our study, caution should be exercised in translating these results into the clinical arena. Previous studies showed that the effects of NMDAR blockade are age- and developmental stage-dependent. It appears that there exists a time window during which the developing brain is exquisitely vulnerable to even transient NMDAR antagonism (Ikonomidou et al. 1999). This “critical period” concept is also supported by other rat studies, in which ketamine administration on gestation day 18 versus day 19 resulted in different behavioral effects in the offspring (Mickley et al. 2001, 2004). In the developing human brain, the time window during which NMDAR blockade is particularly detrimental has not been clearly delineated. This vulnerable period is possibly dictated by the expression pattern and subunit composition of NMDAR in the brain, both of which are incompletely understood in the human fetus (Bagasrawala et al. 2016). Furthermore, the developmental switch in subunit composition from GluN2B to GluN2A may be influenced by maternal ketamine exposure (Zhao et al. 2016). A better understanding in this regard is warranted before obstetric use of ketamine can be considered in clinical practice.

The mechanisms underlying the antidepressant effects of ketamine are probably manifold, targeting a variety of ion channels, cell types, and brain regions (Li et al. 2010; Duman and Duman 2015; Zanos et al. 2016; Cui et al. 2018; Yang et al. 2018; Marwaha et al. 2023). Our analysis has focused on the hippocampus, a region heavily involved in depression and emotional regulation (MacQueen and Frodl 2011). It remains to be examined whether sex differences could also be found in other brain regions involved in the pathophysiology of depression, such as the lateral habenula and prefrontal cortex (Duman and Duman 2015; Cui et al. 2018; Yang et al. 2018), in the offspring of ketamine-treated pregnant mice. On the other hand, although glutamatergic neurons appear to be the major cellular players mediating antidepressant action of ketamine, previous studies have also demonstrated effects of ketamine on GABAergic interneurons and astrocytes (Cui et al. 2018). For instance, it has been reported that prenatal exposure to high-dose ketamine resulted in decreased interneuron density and long-term sex-dependent behavioral effects (Aligny et al. 2014). Whether and how these cells are affected in our model remain to be investigated.

## Conclusions

This study demonstrates that subanesthetic-dose ketamine produces antidepressant-like effects in pregnant mice and their male offspring, without causing overt impairments in locomotion, cognition, or social behavior in the offspring. These findings suggest that the neurological impact of perinatal ketamine exposure may be both dose-dependent and sex-specific, and they offer insights into the developmental origins of emotional regulation. Further investigation is warranted to elucidate the underlying mechanisms, including the roles of glutamate receptor subunit compositions, and explore the potential clinical relevance of these results.

## Supplementary Information

Below is the link to the electronic supplementary material.

Supplementary file1 (PDF 358 KB)

## Data Availability

Data is provided within the manuscript or supplementary information files.
