# Supplementary material for: Sex-Specific Neuropsychiatric Effects of Subanesthetic Ketamine Exposure in Pregnant Mice and Their Offspring
Source: Cell Mol Neurobiol. 2025 Jul 19;45:72. doi: 10.1007/s10571-025-01582-w (PMC12276194; doi:10.1007/s10571-025-01582-w)
Supplement: Supplementary file 1 — Supplementary file1 (PDF 358 KB) [file 10571_2025_1582_MOESM1_ESM.pdf]

Table S1. Oligonucleotide primer sequences.

Primer	Sequence (5’ to 3’)
GluN1-F	TGAGTGATGGCACATGCAAA
GluN1-R	CCCCGTACAGATCACCTTCTTG
GluN2A-F	ACTGGGAAGTTGGACGCTTT
GluN2A-R	ATCTCACCGTCACCAACAAACT
GluN2B-F	TGAGTGAGGGAAGAGAGAGAGG
GluN2B-R	ATGGAAACAGGAATGGTGGA
GluN2C-F	GTGCTTGGACTAGGAAGGTTCT
GluN2C-R	CCCAAGCACCAAGGAGTGAA
GluN2D-F	TCCTGGGGGACGATGAGATT
GluN2D-R	AGTCGCCAGTACACAAGGTG
GluN3A-F	ACGTGTGGAAAAGAGGTCCA
GluN3A-R	ATGTCCTGATGGGTCTGGGT
GluN3B-F	TTGTTTGTCCTGCTGTGCCT
GluN3B-R	GTGGATCTTCTGGCTCGTGT
Tubulin-F	GTCCTTTTGGCCAGATCTTCAG
Tubulin-R	CATCCAAGACAGAGTCAACCAACT
SRY-F	TCTTAAACTCTGAAGAAGAGAC
SRY-R	GTCTTGCCTGTATGTGATGG

The forward and reverse primers used for RT-PCR detection of the listed genes are indicated by “-F” and “-R”, respectively.

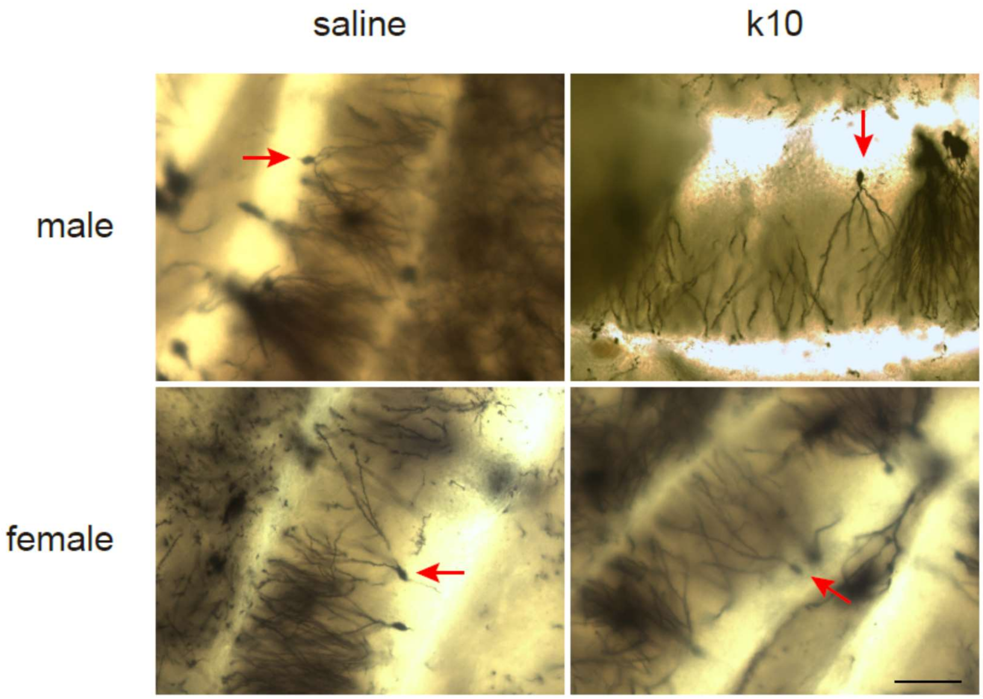

Figure S1. Subanesthetic ketamine treatment during late gestation induces male offspring-specific structural alterations in hippocampal neurons. Representative images of dentate gyrus granule cells, as visualized by Golgi stain (scale bar = 100μm), from male and female offspring of pregnant mice receiving either saline or 10 mg/kg ketamine (k10) treatment daily during gestation day 15-17. The results of further structural analyses are presented in Fig. 3.
